# Role of MiR-204 in controlling metabolic functions of the subretinal microglia

**DOI:** 10.7150/thno.111807

**Published:** 2025-08-11

**Authors:** Yan Chen, Sarah E Bounds, Neloy Kundu, James Regun Karmoker, Yin Liu, Dongin Kim, Jiyang Cai

**Affiliations:** 1 Department of Biochemistry & Physiology, University of Oklahoma Health Sciences Center, Oklahoma City, OK 73104, USA.; 2 Department of Ophthalmology, Dean McGee Eye Institute, University of Oklahoma Health Sciences Center, Oklahoma City, OK 73104, USA.; 3 Department of Neurobiology and Anatomy, University of Texas Health Science Center at Houston, Houston, TX 77030, USA.; 4 Department of Pharmaceutical Sciences, College of Pharmacy, University of Oklahoma Health Sciences Center, Oklahoma City, OK 73117, USA.

**Keywords:** microglia, metabolism, extracellular vesicles, RPE, inflammation

## Abstract

**Rationale:** MicroRNA-204 (miR-204) is one of the most abundant miRNA species in the retinal pigment epithelium (RPE) and RPE-derived extracellular vesicles (EVs). Knockout (KO) of miR-204 leads to dysfunction and degeneration of both the RPE and the retina. In addition to previously reported retinal pathologies, we observed the accumulation of lipid-laden subretinal microglia in miR-204 KO mice. This study aimed to identify potential molecular targets of miR-204 involved in microglia lipid processing and to determine whether RPE-derived EVs can improve the function of miR-204-deficient retinal microglia.

**Methods:** Lipid accumulation in microglia was detected by staining with LipidTox, a fluorescent dye specific for neutral lipids, followed by either flow cytometry analysis or direct visualization on RPE/choroid flat mounts. MiRNA database and target prediction tools, such as miRWalk and TargetScan, were used to search for potential target genes of miR-204 in microglia. The identified target mRNA was validated with a miRNA reporter assay. RPE EVs were prepared from *ex vivo* cultured mice eye cups and administered via retro-orbital injection in miR-204 knockout (KO) mice. RPE integrity was assessed by ERG c-wave measurement.

**Results:** KO of miR-204 resulted in the accumulation of neutral lipids in subretinal microglia. MiR-204 targeted the TGF-β receptor 2 gene in microglia. TGF-β markedly suppressed the expression of genes related to microglia lipid clearance. Eyes injected with RPE-derived EVs showed improved ERG c-wave responses compared to the fellow eyes injected with saline.

**Conclusions:** This study supports that TGF-β/TGF-β receptor 2 regulates microglia lipid metabolism primarily by suppressing lipid clearance. By modulating TGF-β signaling, miR-204 in RPE-derived EVs likely enhances the lipid metabolic activities of subretinal microglia, which are crucial for the structural integrity and proper function of the outer retina and RPE. RPE-derived EVs and their delivery of miRNAs represent a potential therapeutic approach for treating retinal diseases, such as age-related macular degeneration, which involve dysregulated lipid metabolism in subretinal microglia.

## Introduction

MiR-204 is one of the most abundant miRNA species in the retinal pigment epithelium (RPE) 1. Genetic knockout of miR-204 in the RPE reduced the phago-lysosomal activity and suppressed the turnover of phagocytosed photoreceptor outer segments (POS), thereby leading to the progressive RPE degeneration and loss of retinal function 2. Mutation in miR-204 gene causes inherited retinal degeneration and coloboma 3.

In addition to the RPE, retinal microglia also perform phagocytosis function 4. Microglia in the central nervous system can adapt to the microenvironment, displaying plasticity in markers and functions associated with either homeostatic steady-state or activation 5-7. Once in the subretinal space, microglia facilitate the RPE in POS removal 8. The uniqueness of the subretinal microenvironment is an enrichment of the immune regulatory factors released by the RPE 9. RPE-derived extracellular vesicles (EVs), and their associated proteins and microRNAs (miRNAs), can potentially reprogram the subretinal microglia 10. The underlying mechanisms, however, remain elusive. Whether miR-204 can impact microglial function has not been determined.

In the current study, we characterized the retinal microglia in miR-204 KO mice and identified deficiencies in their functions related to lipid clearance. Subretinal microglia in miR-204 KO mice exhibited an accumulation of neutral lipids. We further determined that the TGF-β receptor 2 gene was a target of miR-204. TGF β signaling has profound impact on cell metabolism 11. In microglial cells, TGF β inhibited fatty acid utilization and downregulated genes associated with lipid metabolism. Previously, we reported that miRNAs can be transferred from the RPE to retinal microglia 12. Notably, we found that RPE-derived EVs can improve RPE barrier function in miR-204 KO mice when delivered retro-orbitally. Collectively, our findings suggest that miR-204 plays important roles in regulating retinal microglia functions by modulating their responses to TGF β. RPE-derived EVs have the potential to rejuvenate the protective functions of subretinal microglia, by delivering cargos such as miR-204.

## Material and Methods

### Animals

miR-204 KO mice were provided by Dr. Sheldon Miller's laboratory at the NIH Intramural Research Program 2. Common genetic mutations associated with inherited retinal degeneration, such as *rd1* and *rd8* 13, were screened for and confirmed to be absent in the KO strain. Animal protocols were approved by the Institutional Animal Care and Use Committee (IACUC) of the University of Oklahoma Health Sciences Center (OUHSC), and adhered to the Statement for the “Use of Animals in Ophthalmic and Vision Research” from the Association for Research in Vision and Ophthalmology (ARVO). Mice were housed in the OUHSC animal care facility and maintained on standard rodent chow *ad libitum* under a 12h dark/light cycle.

### Culture of primary retinal microglia

Microglia were isolated from neonatal mice (postnatal days 3-5) and cultured as previously described 12. Cells were cultured in Dulbecco's Modified Eagle/F12 medium (1:1 mixture of DMEM:F12; Mediatech, Manassas, VA) containing 10% fetal bovine serum and 50 ng/mL granulocyte-macrophage colony stimulation factor (GM-CSF) (R&D Systems, Minneapolis, MN). The enrichment of microglia was validated by CD11b staining and flow cytometry, with a typical purity greater than 90% 12.

### Isolation of RPE-derived EVs

Mouse eyes were enucleated from euthanized animals, and extraocular muscles and connective tissues were removed by microdissection. Following retina removal, RPE/sclera/choroid explants were cultured *ex vivo* in serum-free DMEM medium for 4 h. EVs in the conditioned medium were isolated using size exclusion chromatography on a qEV column (Izon Science, Medford, MA), with phosphate-buffered saline (PBS) as the elution buffer. The eluted fractions were examined for the exosome marker proteins TSG101 and flotillin-2 (Flot2) 12, 14. Nanoparticle tracking analyses of particle size and density were performed using a NanoSight NS300 (Malvern Panalytical, Westborough, MA) 12. Ten eye cups were pooled, yielding an EV concentration of approximately 5 x10^8^/mL per eye cup.

### EV treatment and Electroretinography (ERG) c-wave measurement

Under anesthesia, miR-204 KO mice received retro-orbital injection of 100 μL of EV (5x10^8^ particles) per eye. A second injection was administrated 7 days after the initial treatment. The contralateral eye served as a control and received 100 μL of saline at both time points. Seven days following the second injection, animals underwent ERG c-wave measurement 15.

ERG recording was conducted as previously described 16. Briefly, mice were dark-adapted overnight before recording. Anesthesia was induced using ketamine (80-100 mg/kg) /xylazine (5-16 mg/kg). Pupil dilation was achieved with 0.5% Tropicamide (Covetrus, Portland, ME), followed by the application of hydroxypropyl methylcellulose ophthalmic solution. Gold wire electrodes were placed on the corneal surface. ERG a, b and c-wave responses were recorded using a Diagnosys Espion E2 system (Diagnosys LLC, MA, USA) 15.

To track the uptake of EVs by retinal cells, EVs were engineered with the fluorescent BODIPY dye for tracing 17. Poly(ethylene glycol) (PEG) spacers were conjugated to BODIPY-anchors to prevent the EVs from aggregation 17. After retro-orbital injection, the co-localization of BODIPY and IBA-1 staining was examined on RPE/choroid flat mounts.

### RPE/choroid flat mount

Enucleated mouse eyes were fixed in 4% paraformaldehyde (PFA) for 10 min, and extraocular muscles were carefully removed by microdissection. RPE/choroid flat mounts were prepared as previously described 15 and stained with an anti-IBA-1 antibody (Fujifilm Wako, Tokyo, Japan). The total number of subretinal microglia, including both activated and homeostatic resting morphologies, was quantified across the entire wholemount.

### *In vivo* mouse eye examination

Animals were anesthetized by an intraperitoneal injection of a mixture of ketamine (50-75 mg/kg) and xylazine (5-10 mg/kg). The pupil was fully dilated with Tropicamide, and covered with Gonak™ hypromellose ophthalmic demulcent solution (Akorn, Lake Forest, IL, USA). Fundus photographs were taken with a Micron-IV camera (Phoenix Research Laboratories, Pleasanton, CA) with cornea-contacting lens 18. Spectral-domain OCT was performed on a Bioptigen Envisu system (Leica Microsystems Inc, Buffalo Grove, IL, USA) with a mouse retina lens 19.

### 3' UTR reporter assay

The 3′ untranslated region (UTR) target clones for mouse *Tgfbr2* gene, either wild type or mutated, were purchased from GeneCopoeia (Rockville, MD). The construct contains the 3′ UTR of *Tgfbr2* gene spanning positions 2031-4728 (GeneBank sequence ID NM_029575), inserted downstream of the *Gaussia* luciferase reporter gene 20. The mutant construct had nucleotides 2499 and 2500 changed from AA to CC. Primary retinal microglia cultured in 6-well plates were co-transfected with 2.5 μg of the reporter plasmid and 3 pmol of miR-204 mimics or scrambled RNA (Dharmacon, Lafayette, CO), using Lipofectamine 2000 (Invitrogen). Dual-luciferase assays were performed 2 days post-transfection, with *Renilla* luciferase activity, cloned into the same reporter construct 20, used for normalization.

### Cytokine array assay

Conditioned medium from primary retinal microglia were collected from both WT and miR-204 KO cells. Cell debris were removed by centrifugation at 1,000 *g* for 5 min. Samples were applied to membranes in the Proteome Profiler Mouse Cytokine Array Panel A (R&D systems) kit, which can detect 40 different cytokines. Signals were detected with streptavidin-conjugated fluorescent secondary antibody following manufacture's guide.

### Flow cytometry

Mouse RPE/choroid/sclera tissues were collected from enucleated eyes after the removal of the anterior segment and retina. Tissues from six mouse eyes were pooled and digested with 0.2 mg/mL collagenase D (Sigma) and 5 u/mL dispase (Stemcell, Cambridge, MA) at 37 °C for 30 min 15. Cell clumps were removed by passing through a 40-μm cell strainer. The cells were then blocked with an FcγR blocker and stained for cell surface markers using fluorochrome-labeled antibodies: PE-anti-CD45 and Alexa Fluor 700-anti-CD11b (BioLegend, San Diego, CA). Intracellular staining was used for CD68 with PerCP/Cyanine 5.5-conjugated antibody (BioLegend) 15. For neutral lipids staining, cells were fixed in 4% PFA for 30 min and stained with HSC LipidTox Green (Invitrogen, Waltham, MA) at room temperature for 30 min. Samples were analyzed on an Attune NxT acoustic focusing flow cytometer (Invitrogen by Thermo Fisher Scientific, MA). Compensation was performed according to the manufacturer's instructions using UltraComp Beads (Thermo Fisher). Flow cytometry data were processed with Attune^TM^ NxT, FCS Express (DeNovo Software, Pasadena, CA) and FlowJo (Ashland, OR, USA) softwares.

### Seahorse Metabolic assay

Primary mouse retinal microglia were used for the metabolic assays to measure the rates of oxygen consumption and extracellular acidification in the presence of glucose or glucose and palmitate-bovine serum albumin (BSA) 15. 2x10^5^ cells per well were seeded in Seahorse 96-well microplates (Agilent, Santa Clara, CA) and treated with 10 ng/mL TGF-β1 for 16 h. MitoStress and Palmitate Oxidative Stress tests were performed following manufacturer's guides. Normalization was performed with the number of cells per well counted by Cytation 1 (Agilent) after nuclei staining. Data were analyzed in Wave software 15.

### Statistics

Statistical analyses were performed with GraphPad Prism software. Data were analyzed using GraphPad Prism software (Boston, MA, USA). Normality of the data distribution and homogeneity of variance were assessed using the Kolmogorov-Smirnov test and either F-test (two-group comparisons) or the Brown-Forsythe test (for multiple-group comparisons). Between-group differences were assessed by Student's t-test or the Mann-Whitney test, with the level of significance presented as P values. For multiple group comparison, if data passed the normality test (P >0.05), one-way analysis of variance (ANOVA) was used, followed by Dunnett's post-tests (everyone vs ctrl), Turkey's (every group) or Sidak's Multiple comparisons Test (specific pairs). If the data were not normally distributed, the Kruskal-Wallis test (a non-parametric alternative to ANOVA) was used, followed by Dunn's multiple comparisons tests. All data were presented as the means ± SEM.

## Results

**Figure 1:**
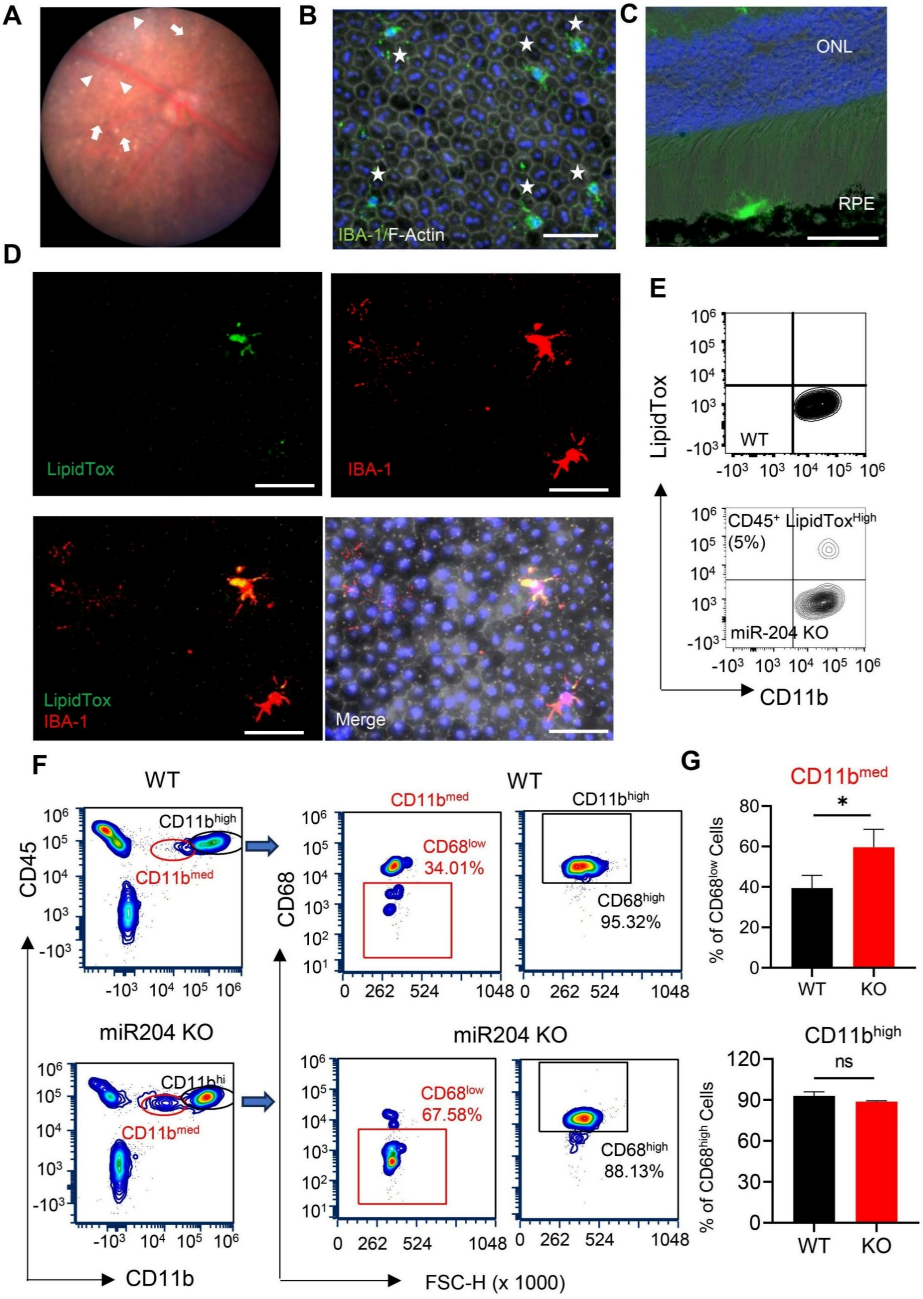
Lipid accumulation in miR-204-KO subretinal microglia. (A) Representative fundus photograph of a 3-month-old miR-204 KO mouse showing RPE pigmentary changes (arrowheads) and retinal deposits (arrows). (B-C) Immunostaining of RPE flat mounts (B) and cryosections of posterior eye tissue (C) from miR-204 KO showing infiltrated microglia at the RPE layer. Subretinal microglia were labeled with IBA1 (Green), and phalloidin (white) was used to delineate the boundaries of the RPE in whole-mount tissues. Blue: Nuclei. ONL: outer nuclear layer. (D) Co-staining of IBA-1^+^ (red) subretinal microglia with LipidTox dye (green) on RPE flat mount prepared from miR-204 KO mouse. (E) Flow cytometry contour plots showing LipidTox and CD11b double-positive cells in RPE/choroid preparations from control and miR-204 KO mice. Scale bar: 50 µm. (F) Flow cytometry of microglia markers, CD11b and CD68, in cells prepared from choroid/RPE fractions of WT and miR-204 KO mice. Quantitation data are presented in (G). Data presented are the averages of 4 separate experiments. * P < 0.05, Student's t-test.

Consistent with previous publications 2, fundus photographs of miR-204 KO mice showed RPE pigmentary changes and retinal cell infiltration at 3 months of age (Figure 1A). Despite these alterations, OCT examination revealed no significant structural changes or retinal thinning in miR-204 KO mice up to 6 months of age (Figure S1A-B). ERG c-wave measurements, an indicator of RPE function, demonstrated an approximately 50% reduction in c- wave amplitude in miR-204 KO mice compared to age-matched controls at 3 months of age (Figure S1C-D). IBA-1^+^ cells with activated morphology were readily detected on the apical side of the RPE on both RPE flat-mounts and cryosections of posterior eyes (Figure 1B-D). Subretinal cells were also observed on H&E-stained paraffin sections (Figure S1E-F). To further investigate lipid accumulation, and we stained microglia with HSC LipidTox dye which measures neutral lipids, including cholesterol and triacylglycerol 21. With flow cytometry assay 22, CD45^med^ cells isolated from RPE/choroid preparations of miR-204 KO mice showed a subset of cells with elevated lipid content (Figure 1E), consistent with findings from the flat-mount imaging. These data suggest that, in addition to its established roles in RPE biology 2, miR-204 KO led to lipid accumulation in subretinal microglia. Lipid accumulation in the RPE and choroid, and the presence of subretinal cells are commonly observed in AMD eyes 23-25. The CD45^med^ cells in miR-204 KO mice showed a higher percentage of cells with lower Cd11b and lower CD68, further support their defects in phagocytosis function (Figure 1F-G) 26.

**Figure 2:**
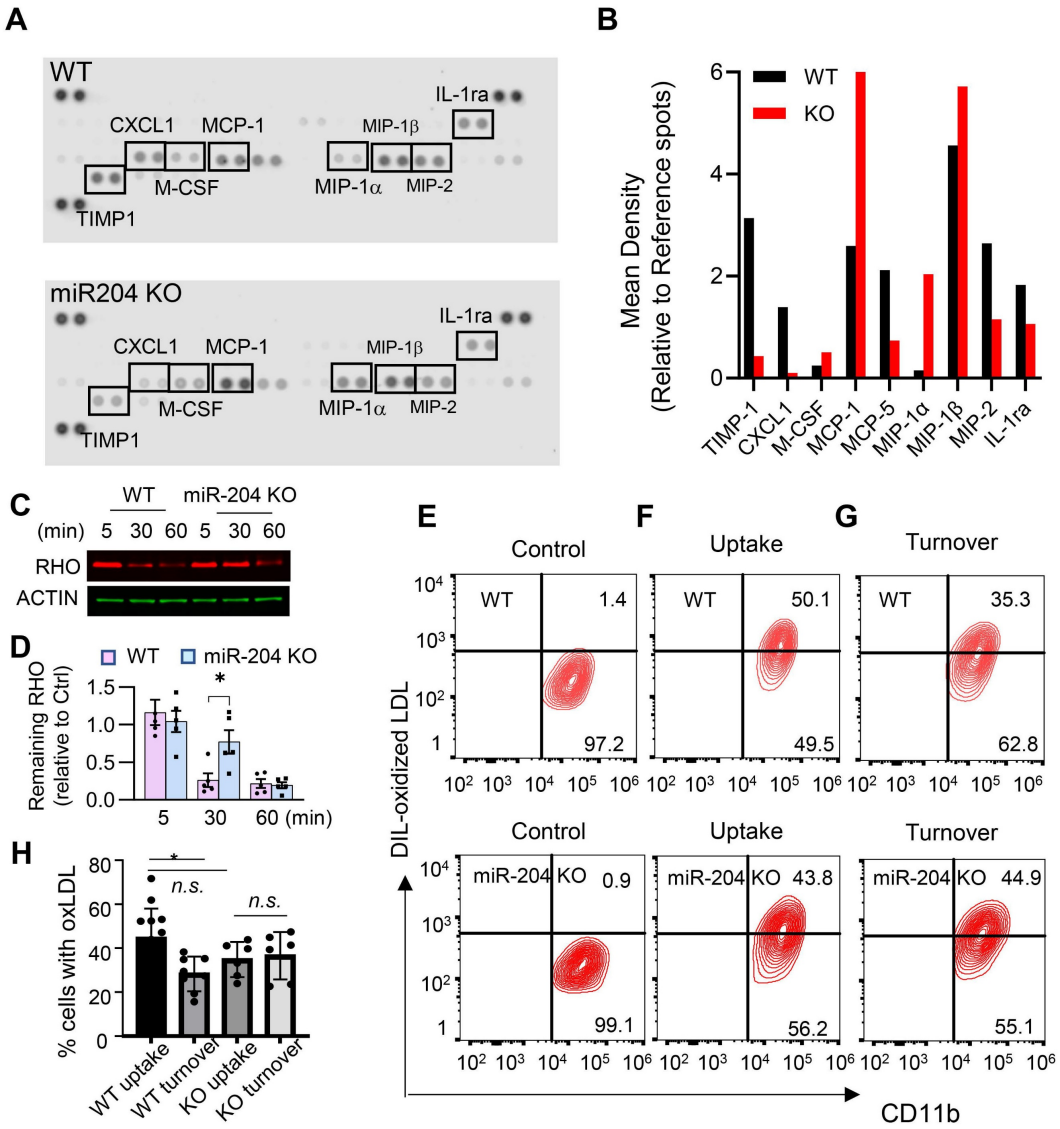
Deficiency in lipid metabolites turnover in miR-204 KO microglia. (A) Cytokine arrays of conditioned media from primary retinal microglia from wild type (WT) and miR-204 knockout mice. (B) Quantitative analyses of cytokine arrays. (C) Western blot analysis of rhodopsin turnover in cultured primary retinal microglia fed with POS. Cells were loaded with POS for 30 min, and rhodopsin levels were monitored at the indicated time points. (D) Quantitative data of rhodopsin turnover normalized to β-actin. (* P < 0.05, one-way ANOVA with Dunnett's multiple comparison post-test, N = 5). (E-G) Flow cytometry analysis of microglia fed with oxLDL. Cells were treated with oxLDL for 30 min (uptake), followed by a 16 h incubation to assess turnover. Quantitative data were presented in (H). Data presented are averages from 4 different experiments (P < 0.05, one-way ANOVA, Dunnett's multiple comparison test).

To investigate lipid metabolism-related functions, cultured primary retinal microglia were used. These cells mainly secreted macrophage chemotactic chemokines (e.g. MCP-1, MIP-1a, and MIP-1b) (Figure 2A and B), and deficiency of miR-204 led to their increased production. Microglia can phagocytose and degrade lipid-enriched POS 4. As shown in Figure 2C and D, WT microglia degraded over 60% of ingested POS within 30 min, as indicated by a reduction in rhodopsin levels (a major protein in POS) to 34 ± 21% of their initial level at 5 min post-loading. In contrast, miR-204-deficient microglia were significantly less effective in degrading POS, with rhodopsin levels remaining at 74 ± 36% of the baseline after 30 min. The differences in POS degradation rates between WT and KO microglia were statistically significant (P < 0.05, Mann-Whitney test, N = 5). We also assessed the ability of miR-204 KO microglia to turn over oxidized LDL (oxLDL), another lipid species microglia can encounter *in vivo* 27. Although both WT and KO microglia exhibited comparable initial uptake of oxLDL (Figure 2E and 2F), KO microglia demonstrated a marked deficiency in oxLDL degradation. Approximately 40% of both WT and miR-204 KO microglia initially contained oxLDL after exposure, the percentage of oxLDL^+^ cells in WT microglia decreased to 28 ± 8% after 16 h of incubation. In contrast, no reduction in oxLDL^+^ cells was observed in miR-204 KO microglia (Figure 2G). The difference in oxLDL turnover between WT and KO microglia was statistically significant (P < 0.05, N = 6, one-way ANOVA, Dunnett's multiple comparison test) (Figure 2H). Together, these results suggest compromised lipid handling ability in miR-204 KO microglia.

**Figure 3:**
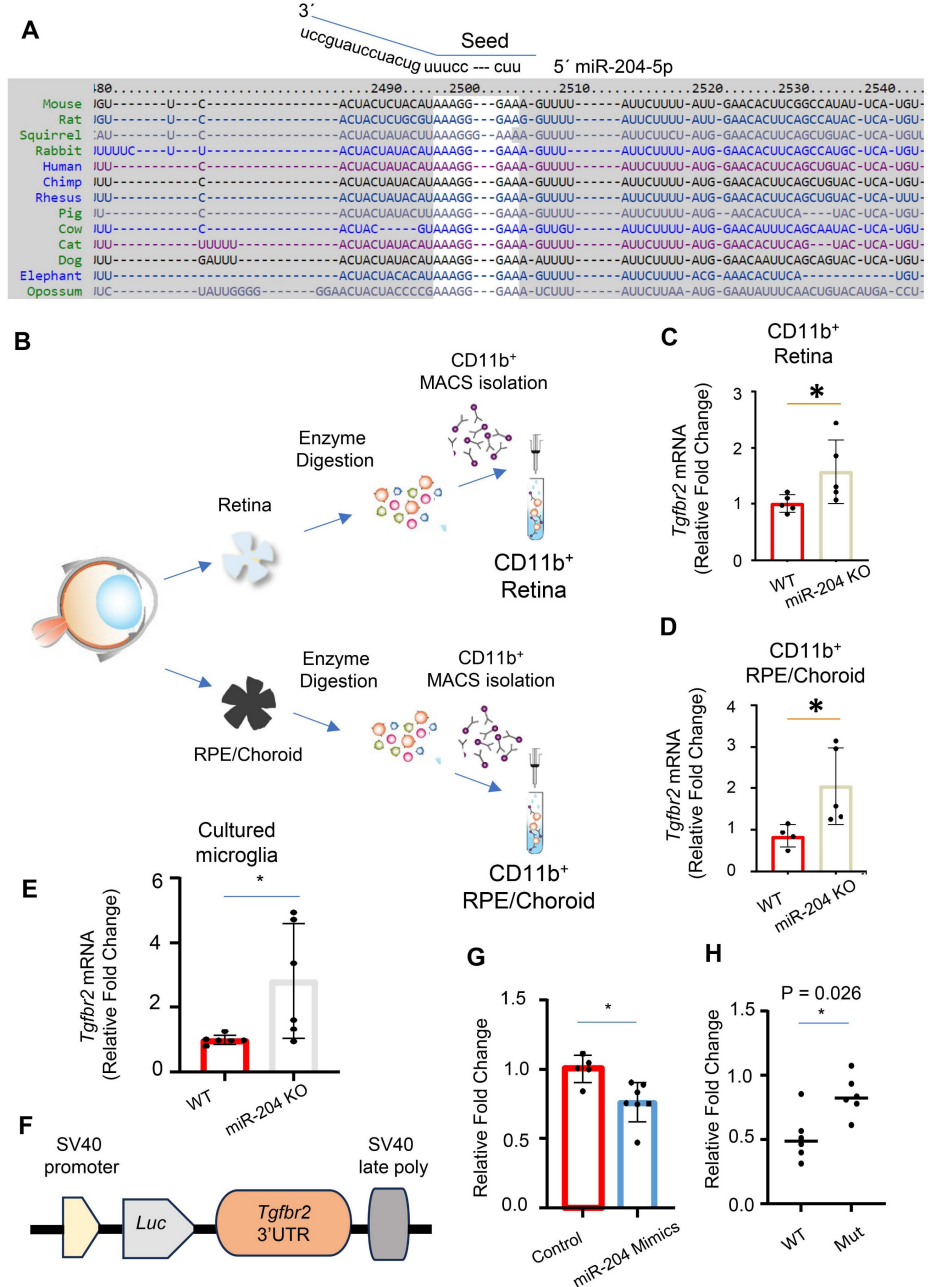
miR-204 directly targets *Tgfbr2* mRNA. (A) Sequence alignment of miR-204 seed sequence with 3′ UTR of *Tgfbr2* mRNA across different species. (B) Schematic representation of the purification of CD11b^+^ cells from retinal and RPE/choroid tissues using MACS isolation. (C)-(E) Quantitative RT-PCR analysis of *Tgfbr2* expression in CD11b^+^ retinal microglia, Cd11b^+^ choroidal myeloid cells, and cultured primary retinal microglia. (F) Schematic illustration of the miRNA reporter assay, with 3′ UTR of *Tgfbr2* mRNA cloned downstream of a luciferase reporter gene. (G) Dual luciferase reporter assay showing reduced luciferase activity following co-transfection of miR-204 mimics with the reporter construct in primary retinal microglia. Data presented are averages from 3 independent experiments. * P < 0.05, Student's t-test. (H) Dual luciferase reporter assay following co-transfection with miR-204 mimics and reporter plasmids containing either the wile-type (WT) or mutated (Mut) *Tgfbr2* 3′ UTR. Data presented are averages from 3 independent experiments. * P < 0.05, Mann Whitney test.

We used miRNA database and target prediction tools, including miRWalk 28 and TargetScan 29, to search for potential target genes of miR-204 in microglia. The 3´ UTR of *Tgfbr2* gene was predicted to have a perfect match with nucleotides 1 - 8 of the miR-204 seed sequence, located at positions 2498 to 2505 (Figure 3A). The pairing is conserved among vertebrates including mouse, rat and primates. To experimentally validate *Tgfbr2* gene as a target of miR-204, we isolated CD11b^+^ cells from WT and miR-204 KO retina or RPE/choroid tissues using magnetic-activated cell sorting (MACS) beads (Miltenyi) (Figure 3B), and used quantitative RT-PCR to measure the level of *Tgfbr2.* The mRNA levels of *Tgfbr2* gene were significantly higher in KO CD11b^+^ microglia and macrophages (Figure 3B -D), as well as in cultured retinal microglia established from miR-204 KO mice (Figure 3E). To confirm the direct interaction between miR-204 and *Tgfbr2* mRNA, we co-transfected microglia with miR-204 mimics or a scrambled control, along with a reporter plasmid containing the 3' UTR of the *Tgfbr2* gene downstream of a luciferase gene (Figure 3F). Binding of miR-204 to the 3' UTR resulted in downregulation of the reporter gene and lowered the luciferase activity (Figure 3G). To validate the specificity of this interaction, we generated a mutant reporter construct by replacing the predicted miR-204 binding site sequence in the 3' UTR (AAAGGGAAAGTTTA) with a mutated sequence (ACCGGGAAAGTTTA) using site-directed mutagenesis. As shown in Figure 3H, the mutant reporter did not respond to miR-204 mimics co-transfection, indicating that the canonical binding site is required for repression. Collectively, these results support that miR-204, the most abundant miRNA species in RPE-derived EVs (Figure S2), directly targets and downregulates *Tgfbr2* in microglia.

**Figure 4:**
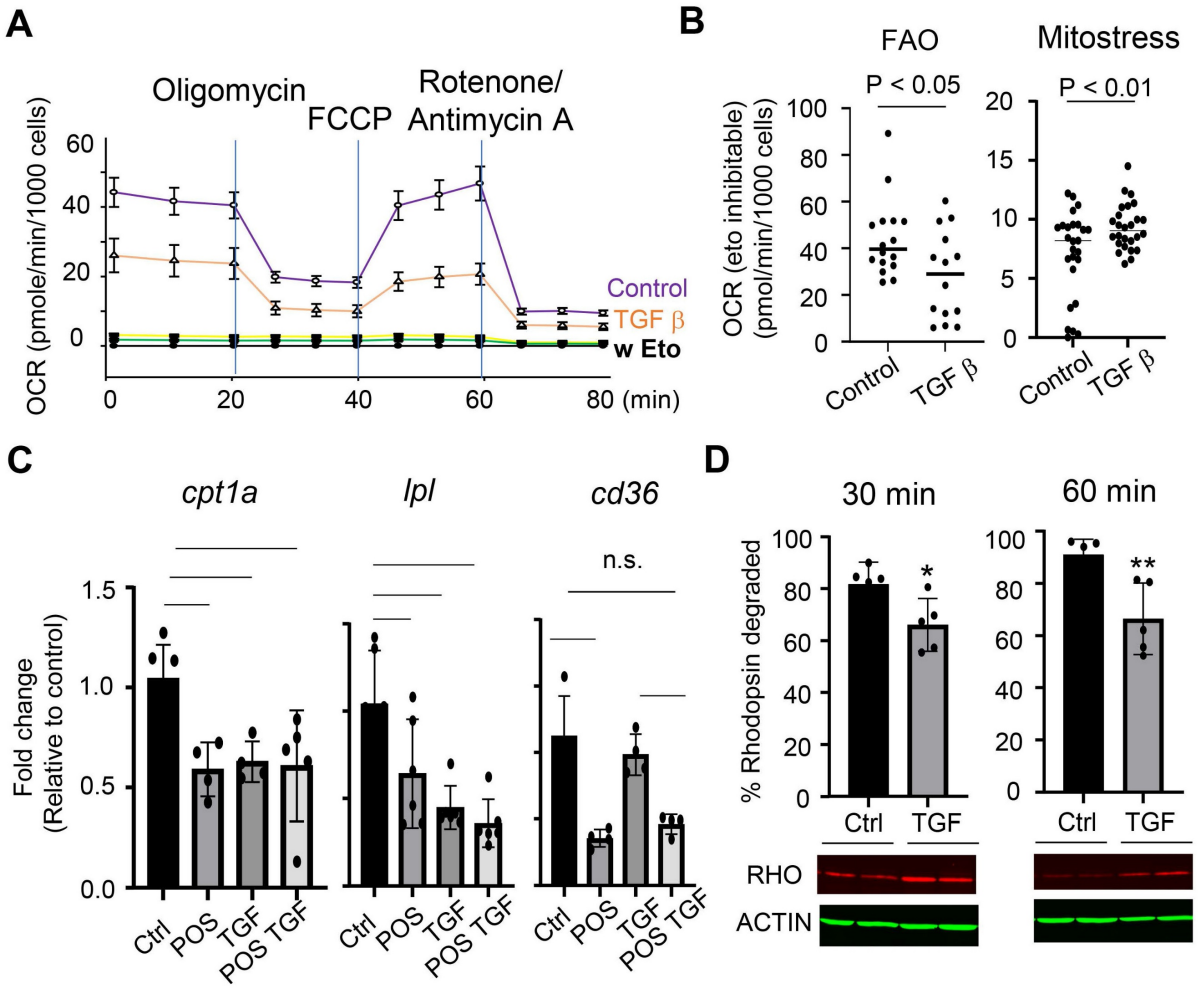
Microglial metabolic shift in response to TGF-β. (A) Control or microglia treated with 10 ng/mL TGF-β for 24 h were subjected to Seahorse Fatty Acid Oxidation assay with palmitate-BSA as substrate for mitochondrial oxygen consumption. (B) Quantitative analysis of oxygen consumption rates from the FAO or Mitostress assay of cells treated with or without treatment of TGF-β. (C) quantitative PCR measurement of *cpt1a, lpl,* and *cd36* gene expression in primary retinal microglia, with or without TGF-β treatment. Data presented are averages from 3 independent experiments (P < 0.05, one-way ANOVA, Dunnett's multiple comparison test). (D) Western blot analysis of rhodopsin turnover in cultured primary retinal microglia fed with POS in the presence or absence of TGF-β. (* P < 0.05, Student's t-test. N = 6).

To further characterize the effects of TGF-β pathway on microglia metabolic activities, we measured glucose- and fatty acid-dependent oxygen consumption rates in cells treated with TGF-β. The results (Figure 4A and 4B) showed that TGF-β treatment reduced fatty acid oxidation (FAO) rates while moderately increased mitochondrial oxidative phosphorylation of glucose. Quantitative RT-PCR analyses showed that TGF-β treatment downregulated the expression of genes crucial for FAO: *Cpt1a* and *Lpl* (Figure 4C). These genes encode proteins that facilitate the transport of long-chain fatty acids into mitochondria 30 and the hydrolysis of triglycerides into fatty acids 31, 32, respectively. The effects of TGF-β on lipid metabolism were selective; C*d36*, which is involved in lipid intake and lipoprotein production and transport, was downregulated by POS treatment (Figure 4C) 33, but remained unaffected by TGF-β treatment (Figure 4C). MiR-204 KO microglia showed reduced basal expression of both* cpt1a* and *lpl*, but their levels were not further decreased in response to TGF-β treatment (Figure 4C). Functionally, TGF-β treatment decreased the turnover rates of rhodopsin in WT microglia (Figure 4D).

**Figure 5:**
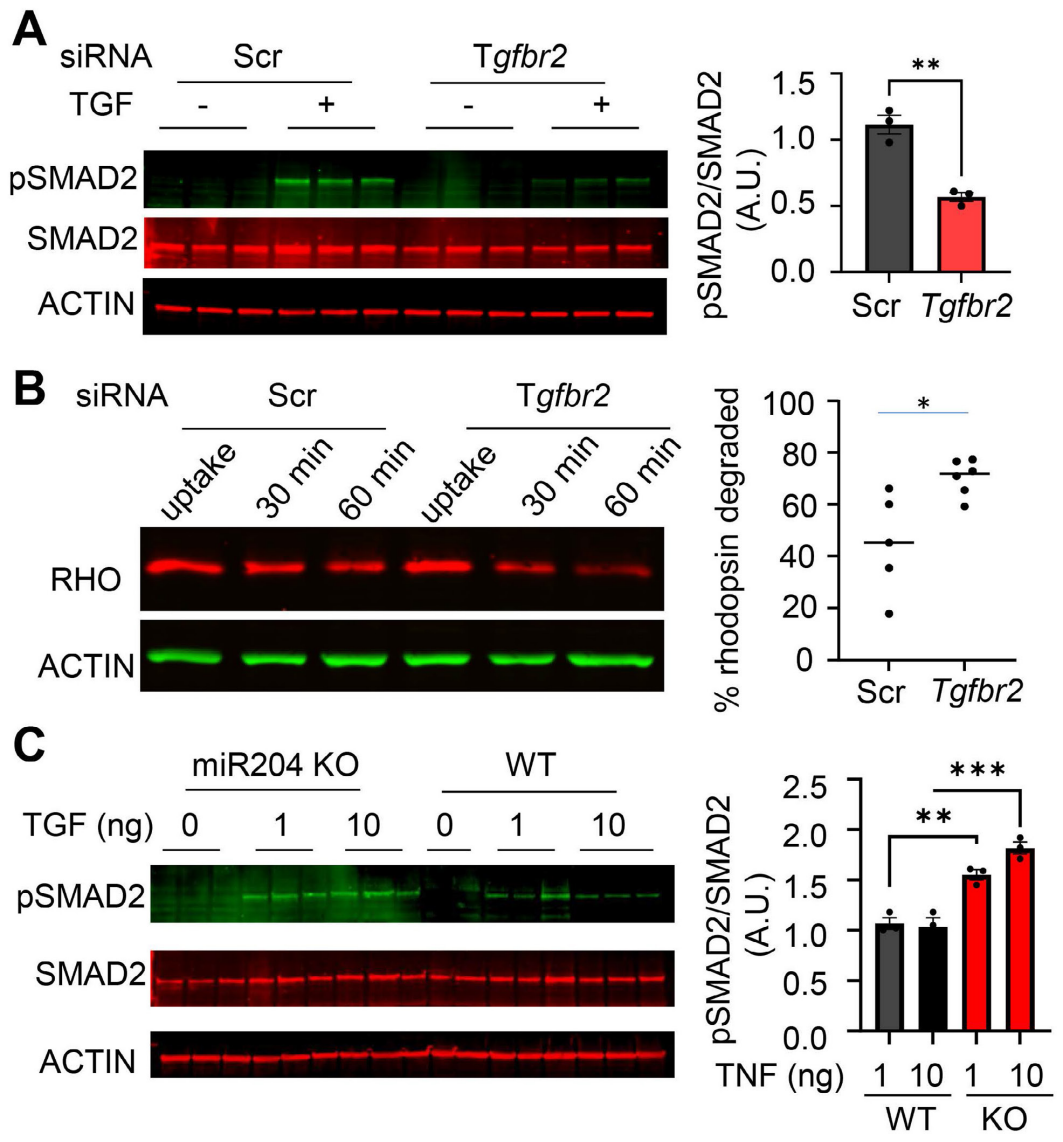
TGF-β signaling modulates POS degradation in retinal microglia. (A) Western blot analysis of SMAD2 phosphorylation in primary microglia transfected with control (Scr) or *Tgfbr2* siRNA and treated with TGF-β (1 ng/mL) (** P < 0.01, Student's t-test. N = 3) (B) Western blot analysis of rhodopsin turnover in primary microglia transfected with Scr or *Tgfbr2* siRNA. Rhodopsin (RHO) degradation was assessed 30 and 60 min after POS uptake (* P < 0.05, Student's t-test. N = 6). (C) SMAD2 phosphorylation in primary microglia prepared from miR-204 knockout (KO) and wild-type (WT) mice treated with increasing doses of TGF-β (0, 1, or 10 ng/mL). (*p < 0.05, **p < 0.01; *p < 0.001; one-way ANOVA with Sidak's multiple comparison test; N = 3).

To validate the functional links, we knocked down *Tgfbr2* in primary microglial cells using siRNA. Transfection with *Tgfbr2* siRNA effectively suppressed TGF-β signaling, as indicated by decreased SMAD2 phosphorylation (Figure 5A). To assess functional consequences, we measured the degradation of phagocytosed POS. *Tgfbr2* knockdown led to an accelerated rate of rhodopsin degradation (Figure 5B), indicating enhanced POS turnover. Consistently, miR-204 KO microglia had heightened response to TGF-β, as reflected by increased SMAD2 phosphorylation (Figure 5C). Together with the impaired POS degradation observed in miR-204 KO microglia (Figure 2), these findings suggest that TGF-β signaling suppresses microglial lipid metabolism. MiR-204 can mitigate the inhibitory effects of TGF-β via targeting *Tgfbr2* gene.

**Figure 6:**
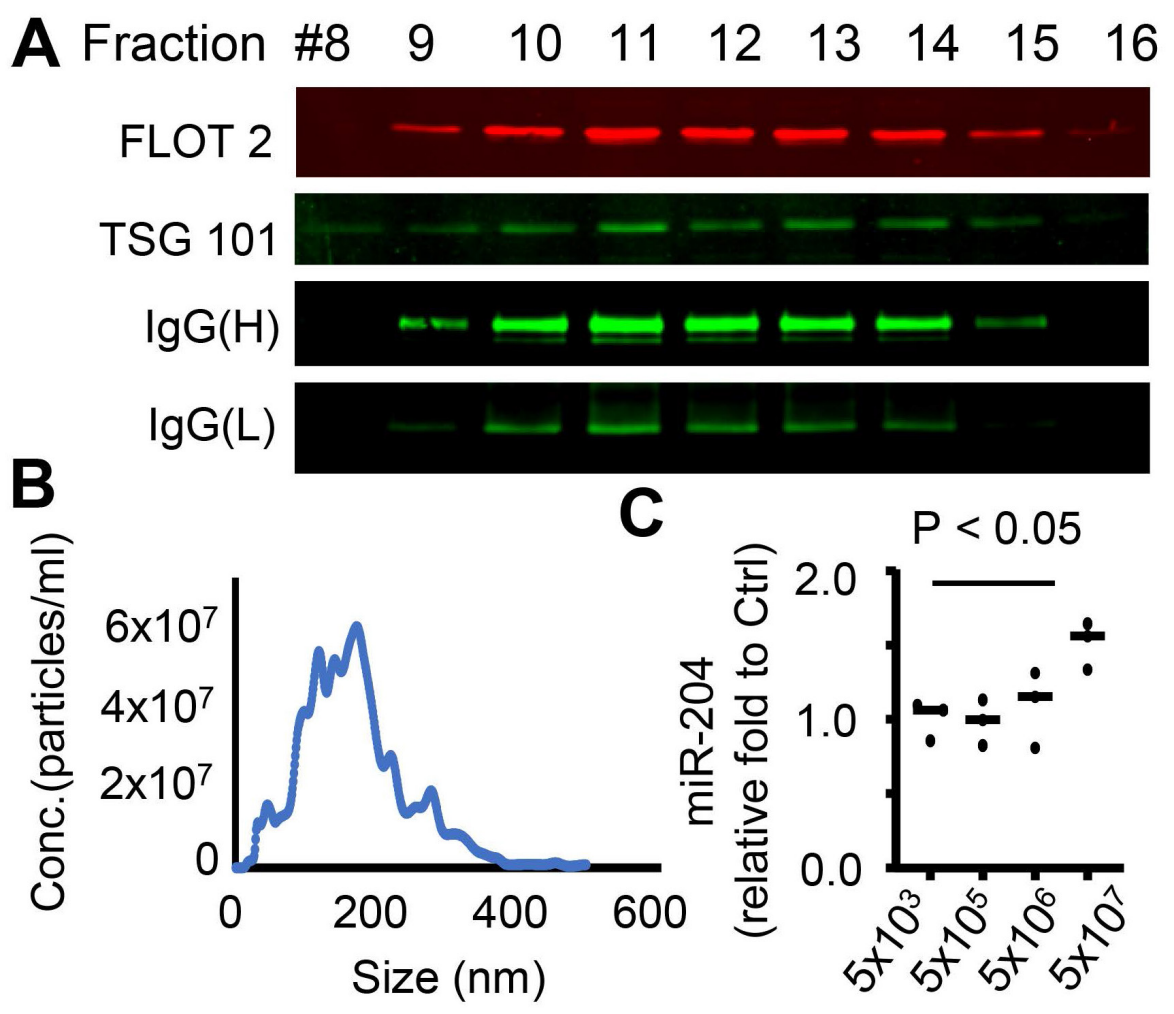
Transfer of miR-204 via RPE-derived EVs. (A) EVs were purified from RPE-conditioned medium through size fractionation. Western blot analysis shows enrichment of EVs markers in selected fractions. (B) Nanosight tracking analysis measured the density and size distribution of purified EVs. (C) Quantitative RT-PCR assessed the level of miR-204 in WT microglia treated with indicated amounts of RPE-produced EVs. Data presented are averages from 3 independent experiments (P < 0.05, one-way ANOVA, Dunnett's multiple comparison test).

We have previously demonstrated that EV can mediate the transfer of miRNAs between the RPE and microglia 12, potentially serving as a signaling mechanism between these cells. MiR-204 is the most abundant miRNA species in RPE-derived EVs (Figure S2). To confirm the transfer of the miR-204 from the RPE to microglia via EVs, we employed an *ex vivo* RPE culture that rapidly released EVs into the conditioned medium (CM) 12, 34, 35. The RPE/eyecup-CM was fractionated by size exclusion chromatography on a qEV column (Izon Science), and the eluted fractions were examined for the presence of exosome marker proteins TSG101 and FLOT2 36, 37. A total of 28 fractions were collected, with EV-enriched fractions identified from 9 to 15 (Figure 6A). Using nanoparticle tracking analyses 13, we quantified that the concentration of EVs was approximately 5x10^8^/mL per eye cup preparation (Figure 6B). These purified RPE EVs were then applied to cultured retinal microglia. Quantitative RT-PCR (Figure 6C) was used to measure the relative level of miR-204 in microglia received EVs. Endogenous level of miR-204 in microglia was significantly elevated by RPE EVs treatment.

**Figure 7:**
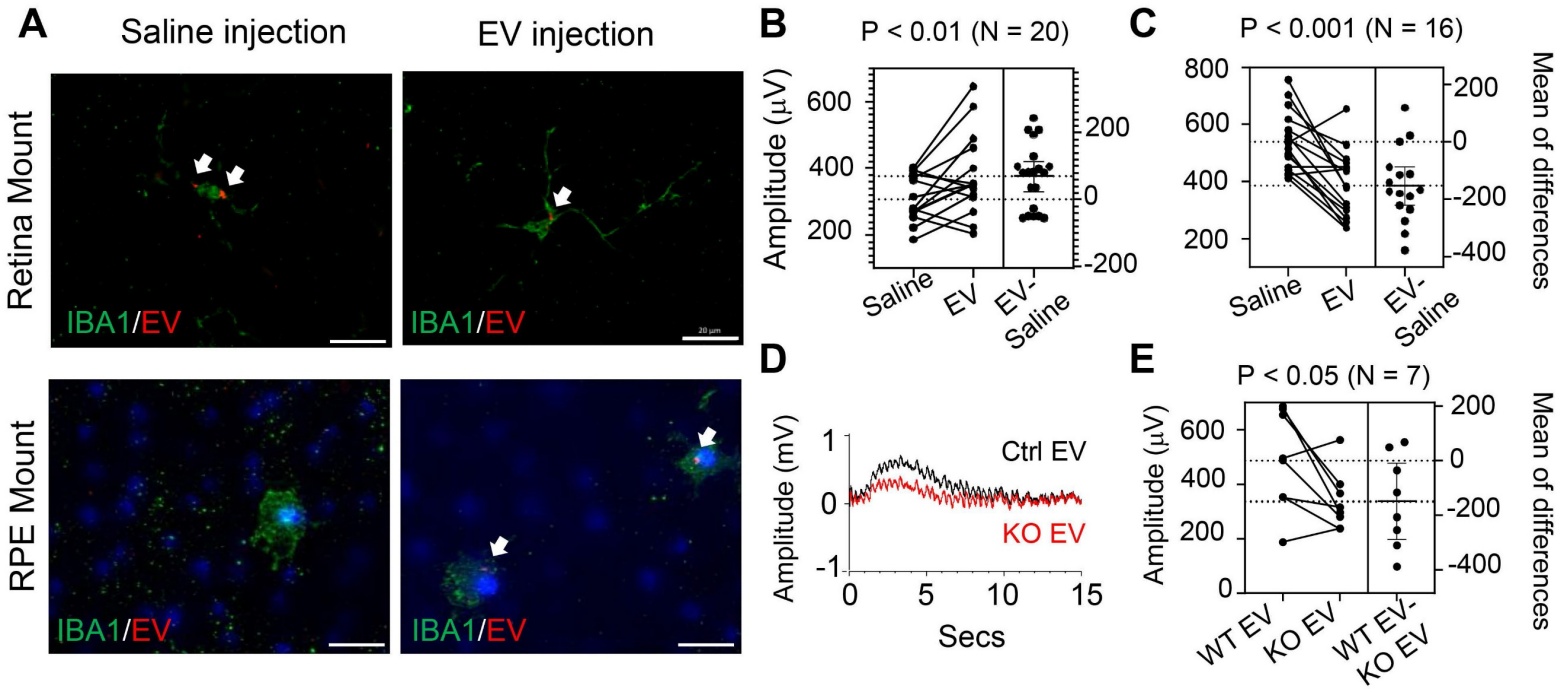
*In vivo* delivery of RPE EVs by retro-orbital injection. (A) Immunostaining of IBA1^+^ cells in retina or RPE flat mount from mice with RPE-derived EV injections. White arrows mark EV-containing microglia. (B) and (C) ERG c-wave responses in eyes of miR-204 mice after retro-orbital injection of RPE-derived EVs from wild-type animals. Data presented are comparisons between contralateral eyes from the same animal receiving EVs or Saline. Paired t-tests were used to assess the differences in c-wave responses after EV treatment, with the number of tested animals indicated on the figure. (D) Representative ERG c-wave traces from an animal receiving WT RPE-derived EVs (Ctrl) and miR204 KO RPE-derived EV (miR204 KO) in contralateral eyes. (E) Estimation plot of ERG c-wave amplitudes in animals receiving WT and miR204 KO RPE-derived EVs in contralateral eyes. Paired t-tests, N = 7.

To assess the impacts of RPE-derived EVs on retinal function and structural integrity, we performed retro-orbital injections of EVs into miR-204 KO mice. Each eye received 5x10^8^ EV particles in a 100 µL volume, while the contralateral eye received a saline injection as a control. The procedure was repeated one week after the initial injection. The dose is consistent with previously reported dosages used for intraocular injection 38, 39. ERG c-wave measurement was carried out 7 days after the second injection. The results showed that EV injection effectively improved c-wave responses in eyes when contralateral control eyes had c-wave amplitudes below 400 µV (Figure 7B). However, in instances where control eyes were relatively healthier with c-wave amplitudes exceeding 400 µV, EV treatment reduced the RPE barrier function (Figure 7C). The protective effects were diminished in animals receiving EVs prepared from miR-204 KO mice (Figure 7D and E), supporting the essential roles of miR-204s for the protective functions of RPE-derived EVs. Although EVs delivered by retro-orbital injection to one eye can reach the retina of the contralateral side, their targeting of subretinal microglia was restricted to the injected side (Figure 7A). The outer retina is avascular. The delivery of EV to the subretinal microglia was likely achieved by local diffusion system. This is similar to selective targeting of a specific population of retinal cells reported with retro-orbital injection of adeno-associated virus 40.

**Figure 8:**
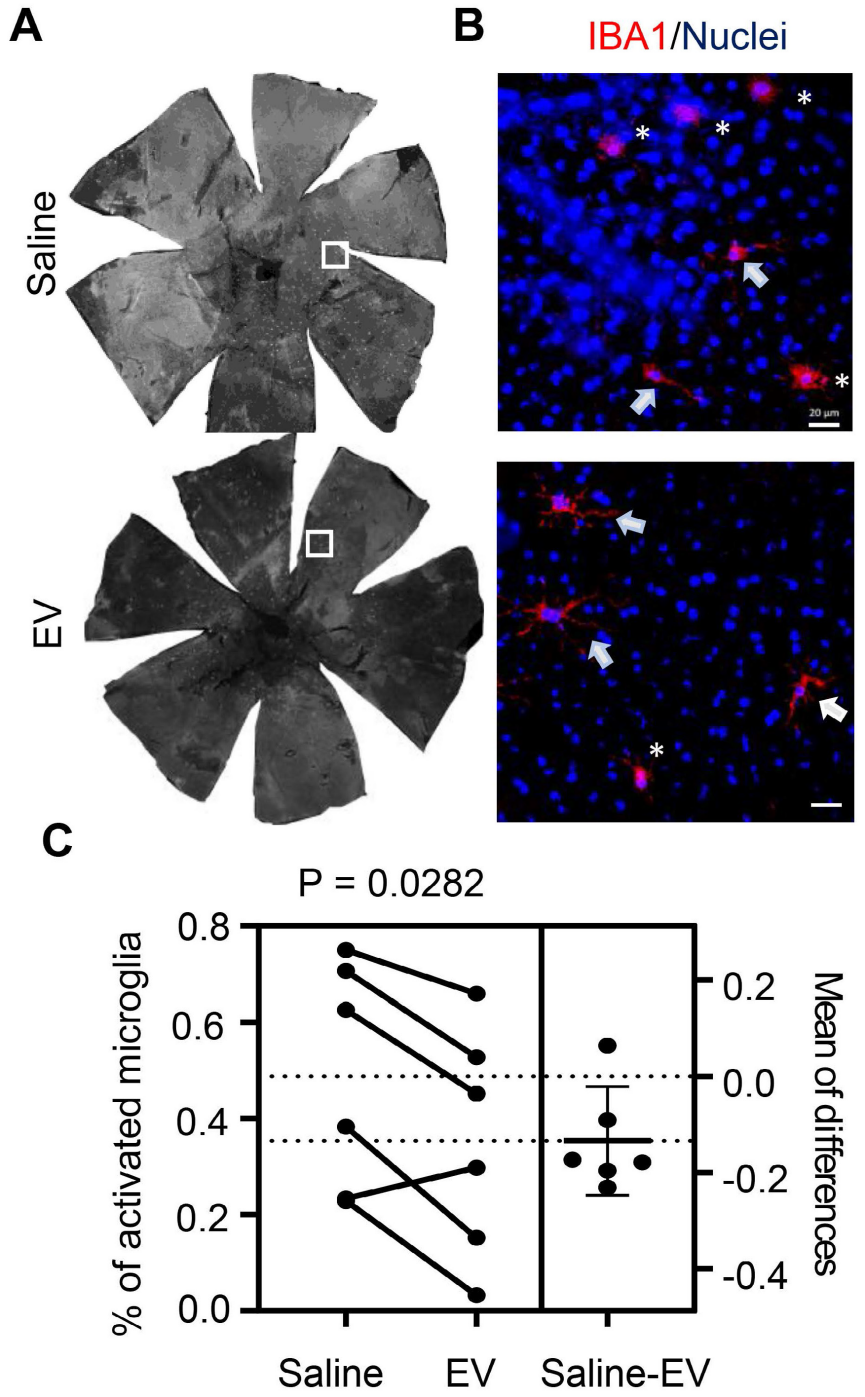
Quantitation of IBA1-positive subretinal microglia after treatment with RPE-derived EVs. (A) Representative images of IBA1-positive cells in whole RPE/choroid mounts from a miR-204 KO mouse at 4 months of age, after receiving EV injection in one eye and saline solution in the contralateral eye. (B) Higher resolution images of the areas boxed in panel A. Arrows indicate resting microglia; asterisks denote activated microglia. Red: IBA1; Blue: Nuclei. Scale bar: 20 µm. (C) Estimation plot showing quantitative results of the percentage of activated microglia under each treatment condition. P value was determined by paired Student's t-test (N = 6).

Upon examining the effects of EV treatment on subretinal microglia, we observed a decrease in the percentage of cells with activated morphology compared to those in saline-treated contralateral eyes (Figure 8). Histopathological examination found no difference in eyes with and without EV treatment (Figure S3A). Scotopic and photopic ERG measurements did not identify differences in photoreceptor cell functions after EV treatment (Figure S3B). Retinal thicknesses, as revealed by OCT scan, were not affected by EV (Figure S3C). Thus, EV treatment appears particularly beneficial for eyes with pre-existing RPE injury, likely by promoting inflammation resolution through enhanced lipid metabolic functions in subretinal microglia.

## Discussion

In retinal diseases with chronic and low-grade inflammation, such as AMD 41-43, parenchymal microglia migrate from plexiform layers into subretinal space to assist in the removal of debris from the photoreceptor cells and RPE 4. Subretinal microglia are exposed to a high load of lipid metabolites. However, the mechanisms that preserve their physiological and protective functions of microglia while preventing their overactivation remain largely elusive. The RPE actively participates in immune modulation 44. MiRNAs encapsulated within RPE-derived EVs can be transferred to microglia 12, acting as a signaling mechanism that regulates gene expression in subretinal microglia.

The intraocular environment is known for its immunosuppressive nature 45, where growth factors and cytokines like TGF-β can dampen both adaptive and innate immune responses 46. Subretinal microglia, critical for clearing lipid metabolites from the RPE and photoreceptor cells 47, encounter inhibitory factors as they migrate from the inner retina. These factors suppress their metabolic activities, resulting in ineffective inflammation resolution. MiR-204, the most abundant miRNA species in the RPE and RPE-derived EVs 1, increased in microglia following the treatment with RPE-derived EVs (Figure 6C). One of the targets of miR-204 is *Tgfbr2*. By downregulating this receptor, RPE EVs can alleviate the inhibitory impact of TGF-β on subretinal microglia, which impedes lipid turnover (Figure 2) and utilization (Figure 4). The effects of TGF-β on cellular metabolic functions have been studied extensively in various types of cells and usually are context dependent. In cancer cells and epithelial cells, TGF-β often induces epithelial-mesenchymal transition (EMT) and mitochondrial dysfunction 48. TGF-β can also regulate many glucose metabolizing enzymes via SMAD-independent non-canonical pathways 11. In macrophages and dendritic cells, TGF-β promotes lipid droplets (LD) accumulation 49, 50. The detailed molecular pathways underlying these metabolic effects have not been established. One of our future studies will be to overexpress TGFBR2 in microglia cells and explore the downstream signaling events. With our flow cytometry assays and LipidTox staining, we observed a relatively small percentage of the CD11b+ cells with lipid accumulation (Figure 1E). Such findings suggest that subretinal microglia, rather than the homeostatic macrophages in the choroid 51, are responsible for handling the lipid waste generated by the photoreceptor cells.

When delivered by retro-orbital injection, EVs can quickly reach the retina 52 and are taken up by microglia (Figure 7A). EVs injected into one side of the eye can traverse the other side via systemic circulation, a phenomenon also demonstrated with AAV-mediated, cell type-specific gene expression 40. However, subretinal microglia, which presumably do not receive supplies from systemic circulation, can uptake these injected EVs through local perfusion. The response between the two eyes were observed to be independent (Figure 7B). Using the contralateral eye as a control, we found that EVs effectively improved ERG c-wave responses only in eyes with existing defects in RPE barrier. Activated myeloid cells have upregulated Fcγ receptors 53. Similar to previously reported IgG^+^ exosomes from other sources 54, 55, RPE-produced EVs contain IgG (Figure 6A), which can facilitate the binding to microglia and improves the specificity of cargo delivery. In addition to microglia, RPE can also engulf the EVs. Our study cannot rule out the contribution of RPE engulfed EVs to the protective effects.

A significant challenge in developing EV- and miRNA-based therapies arises from the intricate nature of miRNA-mRNA interactions. A single miRNA species can target several hundred different mRNAs 56; and it is common for multiple miRNAs to cooperatively target the same gene or genes within the same pathways 57, 58. EVs in RPE-CM carry numerous miRNA species 12. Effective interpretation of these “Big Data” requires the development of sophisticated computational methods to study the effects of miRNAs at the genome-wide level.

In conclusion, our studies revealed an important role of miR-204 in regulating retinal microglia lipid metabolism functions. Transferring miR-204 via EVs represents a potential mechanism for intercellular communication between the RPE and subretinal microglia, contributing to a protective immune response following RPE injury.

## Supplementary Material

Supplementary figures.

## Abbreviations

CM: Conditioned Medium
DMEM: Dulbecco's Modified Eagle Medium
ERG: Electroretinography
EV: Extracellular Vesicle
FAO: Fatty Acid Oxidation
Flot2: Flotillin-2
KO: Knockout
miRNA: microRNA
POS: Photoreceptor Outer Segments
RPE: Retinal Pigment Epithelium
3′ UTR: 3′ Untranslated Region
