# Supplementary material for: Role of MiR-204 in controlling metabolic functions of the subretinal microglia
Source: Theranostics. 2025 Aug 11;15(17):8952–63. doi: 10.7150/thno.111807 (PMC12439270; doi:10.7150/thno.111807)
Supplement: Supplementary file 1 — Supplementary figures. [file thnov15p8952s1.pdf]

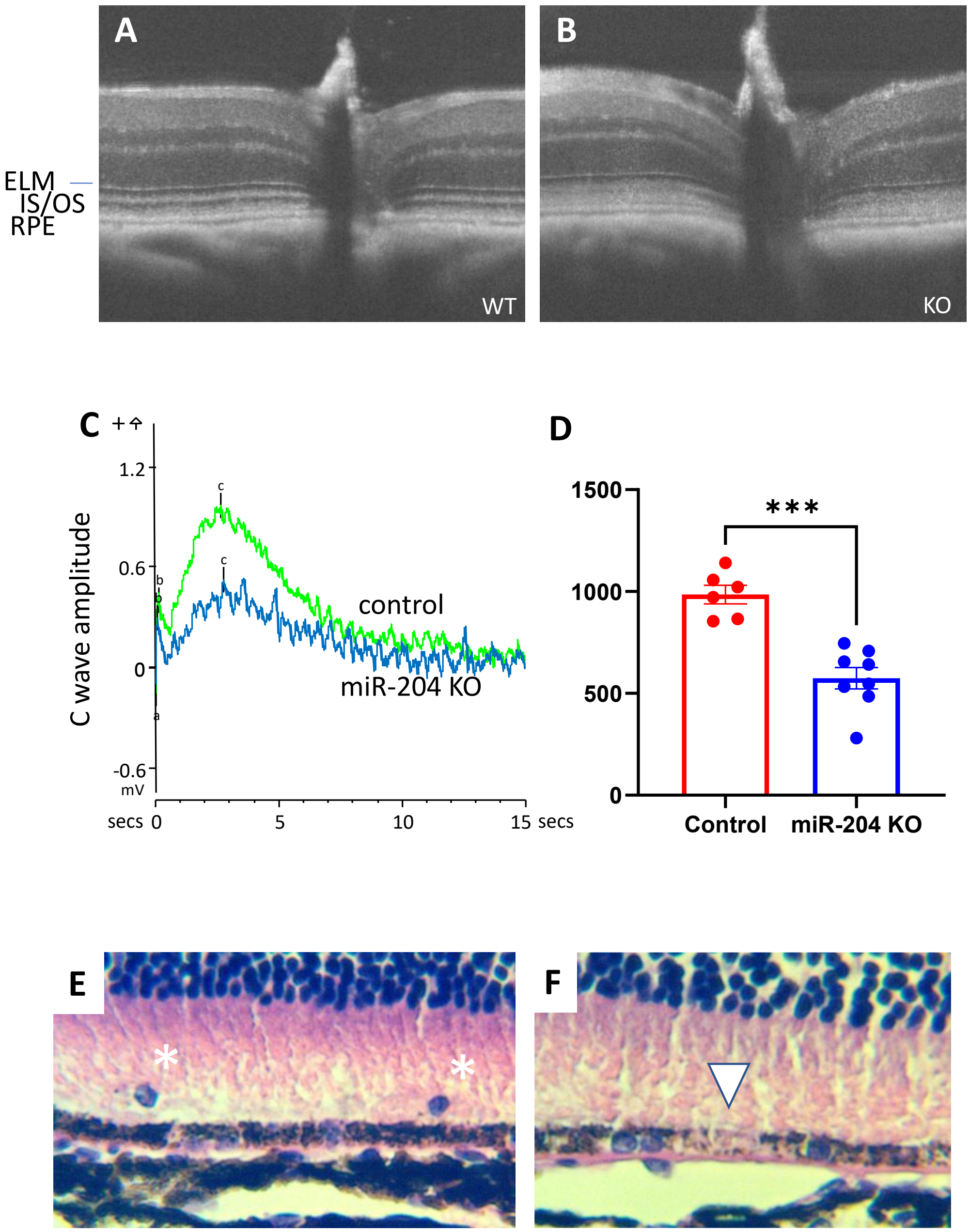

Fig S1. Ocular phenotype of miR-204 KO mice. (A) and (B) OCT scan of a 6 month-old KO mouse. (C) and (D) ERG-c-wave measurement from KO mice at 3-5 months of age. Data presented are averages from 7 animals (*** P<0.001, Student's t-test). (E) Histopathology on paraffin sections of miR-204 KO mice at 3 months. * subretinal cell infiltration. Arrow head: RPE pigment loss.

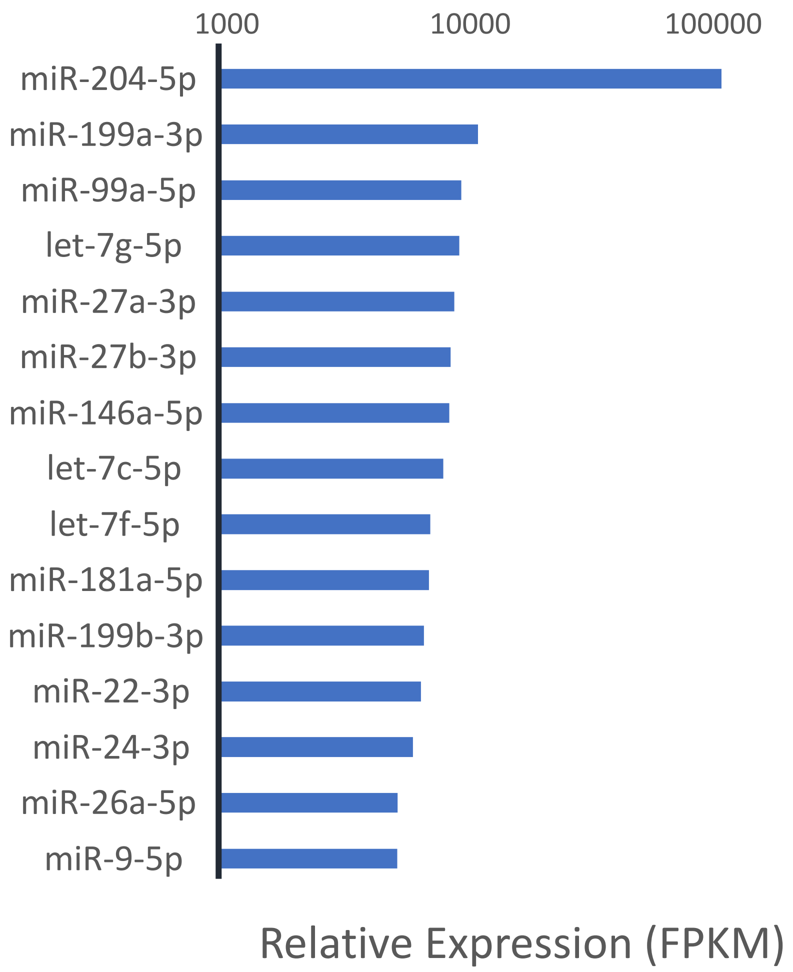

Fig S2. Top 15 miRNA species in RPE EVs. Bulk RNA sequencing was performed on RPE EVs. The miRNA species with the top 15 highest fragments per kilobase million (FPKM) were presented.

Fig. S3

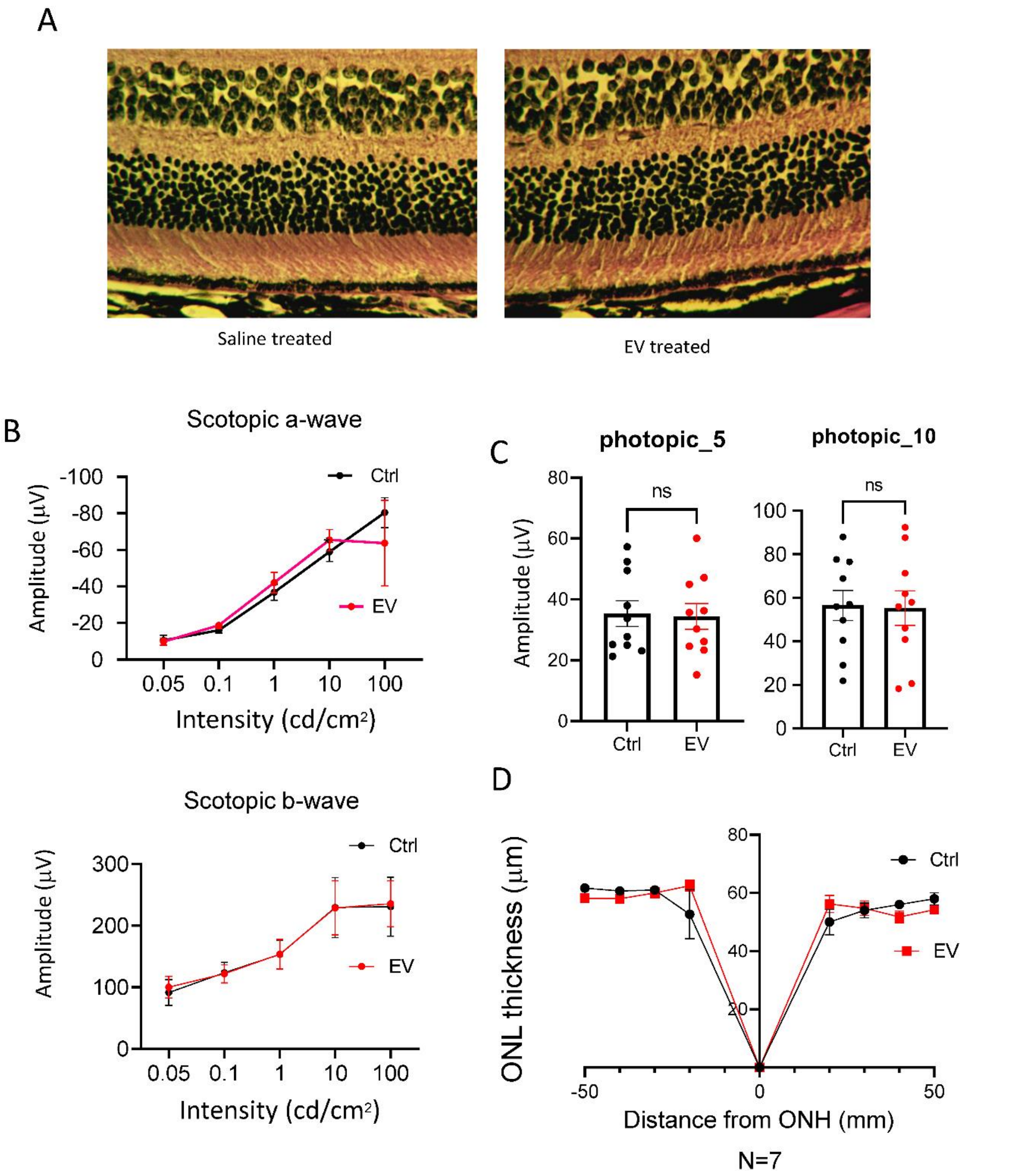

Fig. S3. Effects of EV treatment on retinal structure. (A) H&E stained paraffin sections from eyes of a 2.5 month-old miR-204 KO mouse, with and without EV treatment. (B) and (C) ERG measurements taken at indicated flash light intensities. (D) Retinal thickness measurements from OCT scan of 204 KO mice with and without EV treatment.

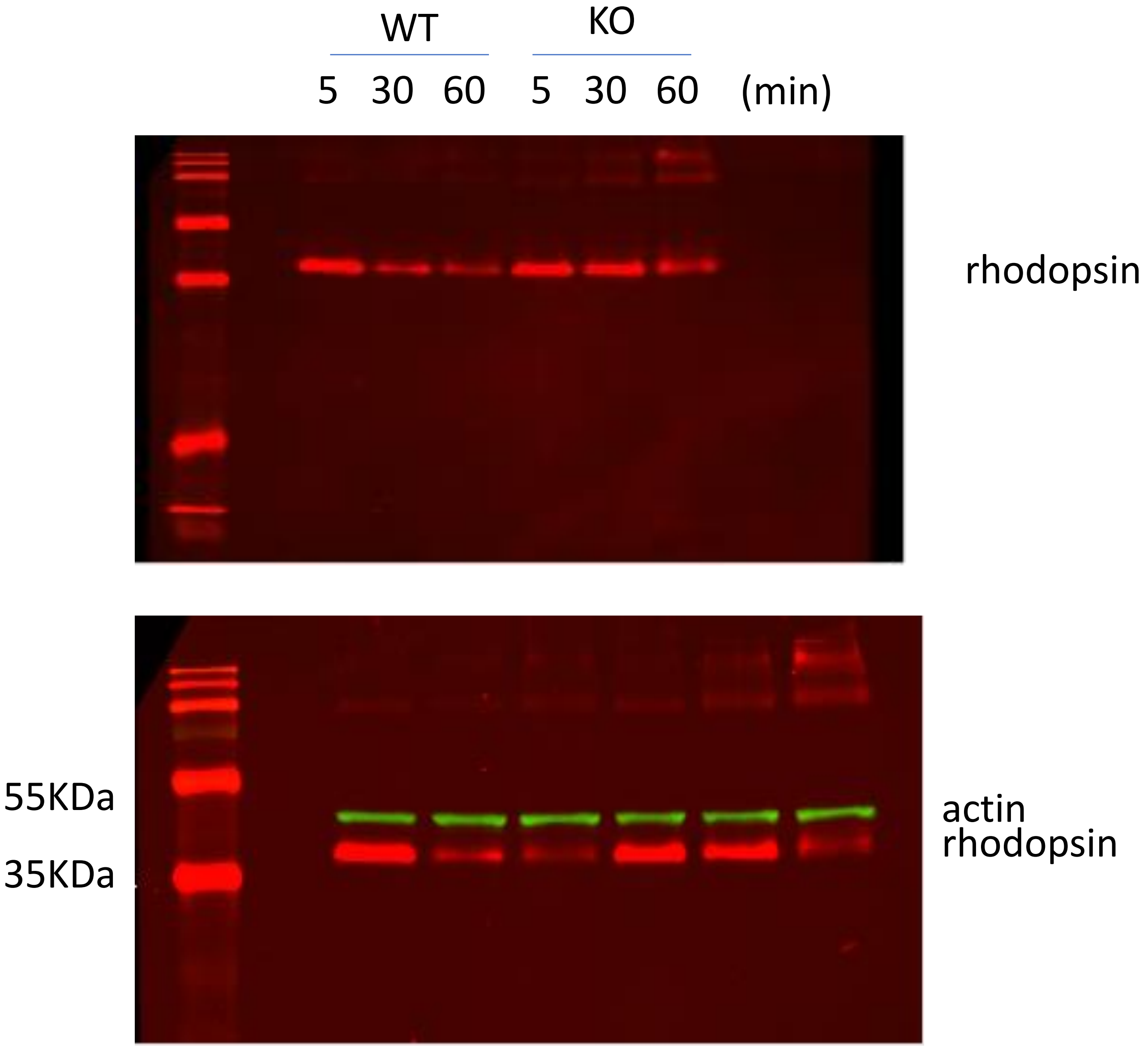

Fig 3C original scan

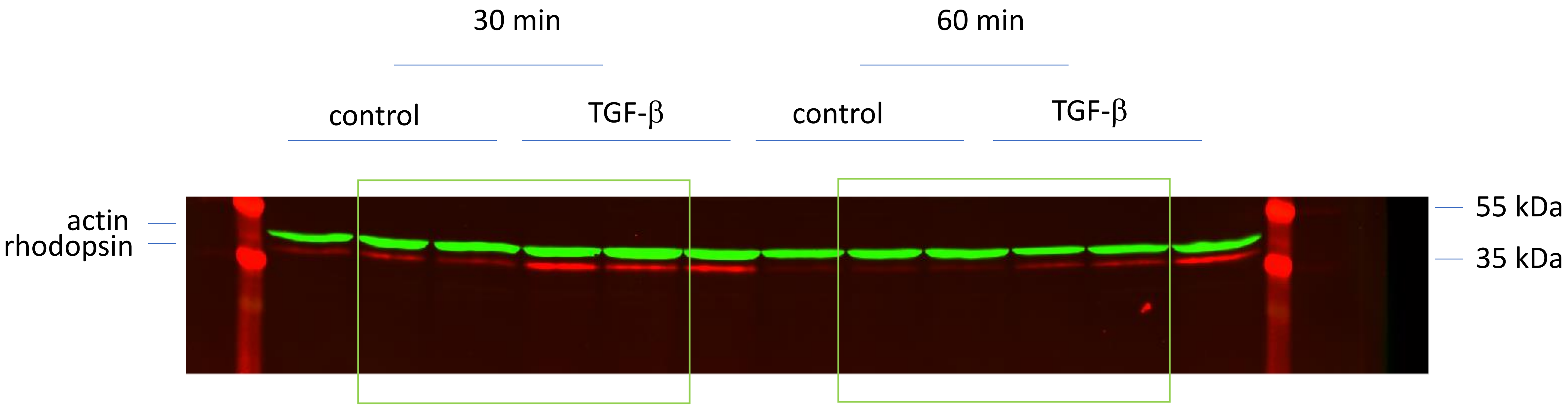

Fig 4D original scan

Fig 5A original scan

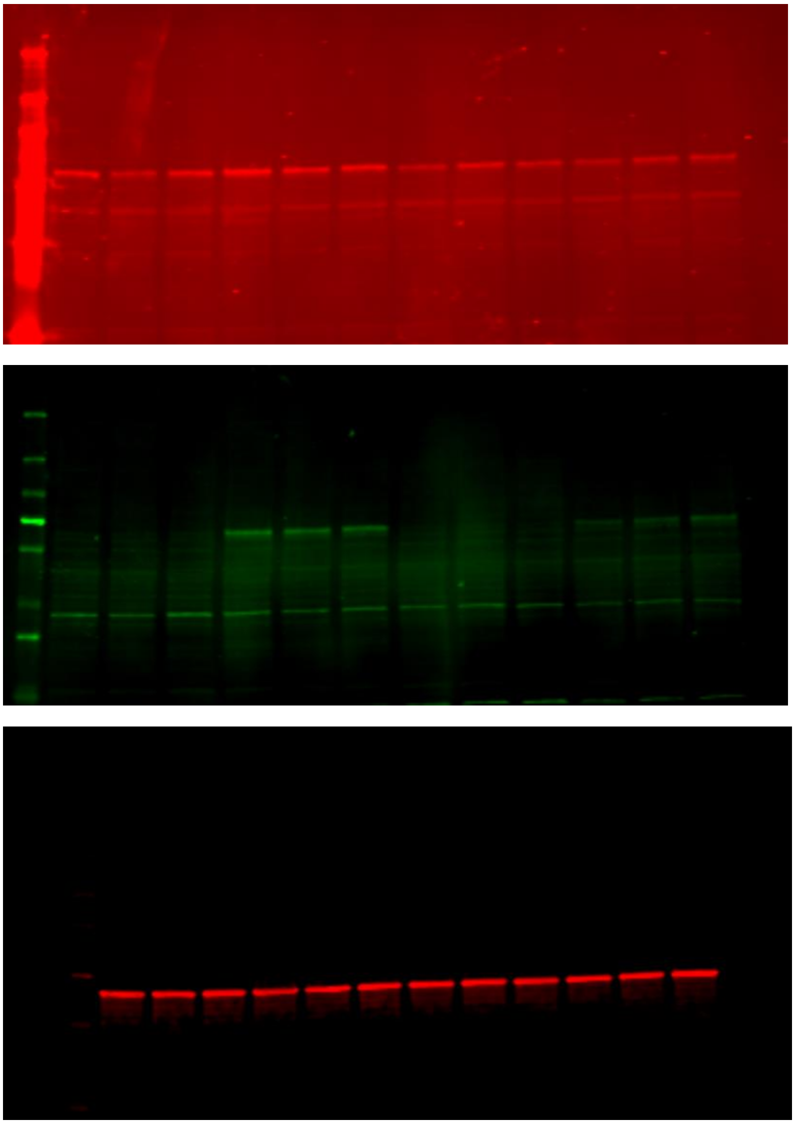

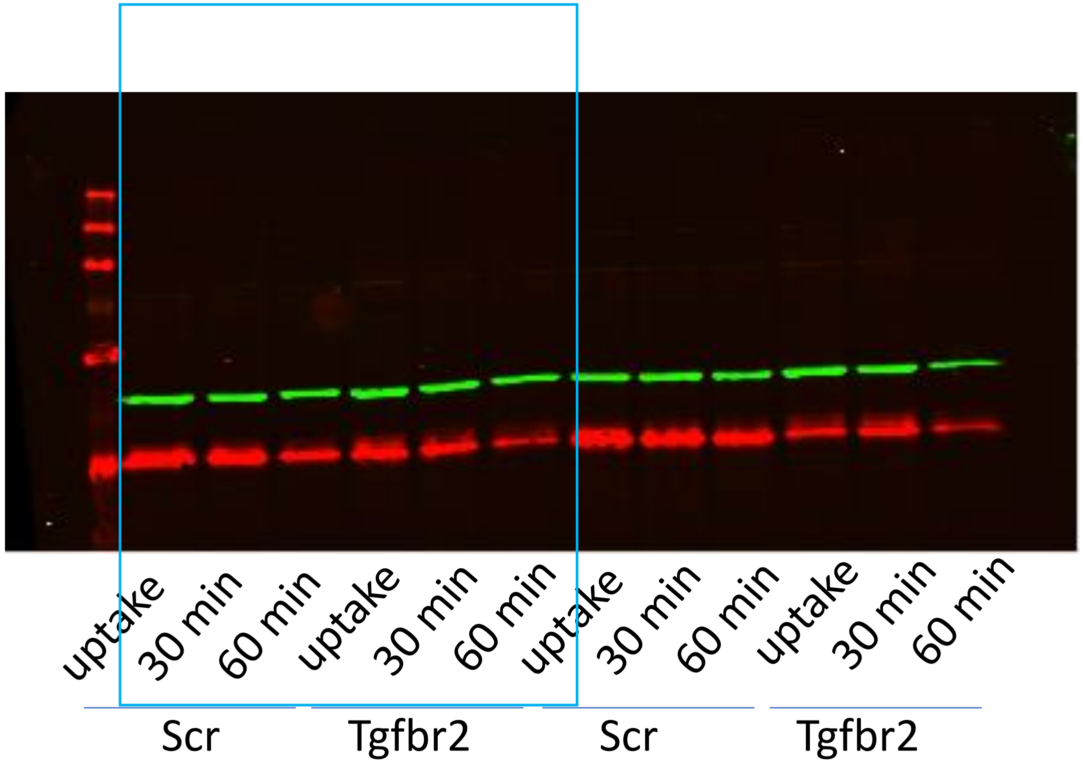

Fig 5B original scan

Fig 5C original scan

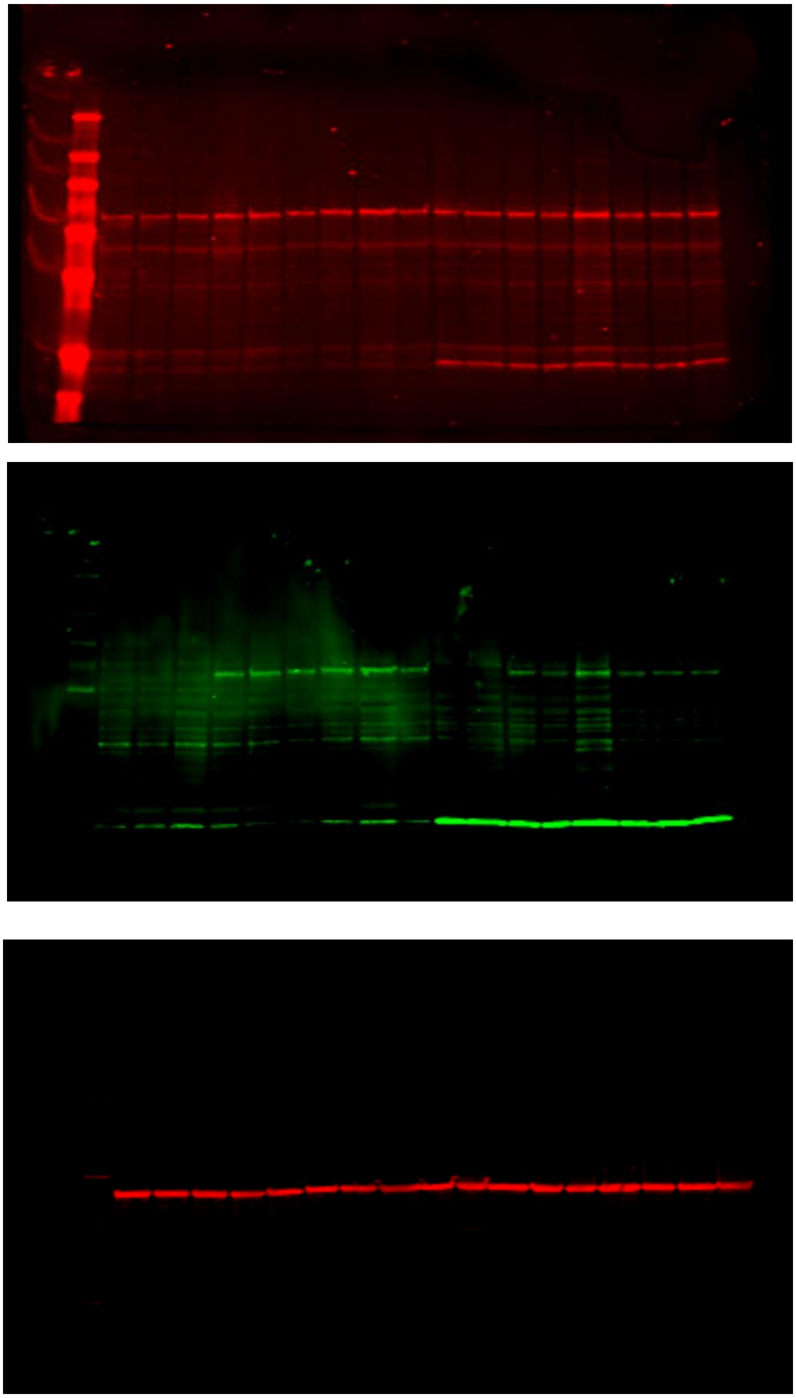

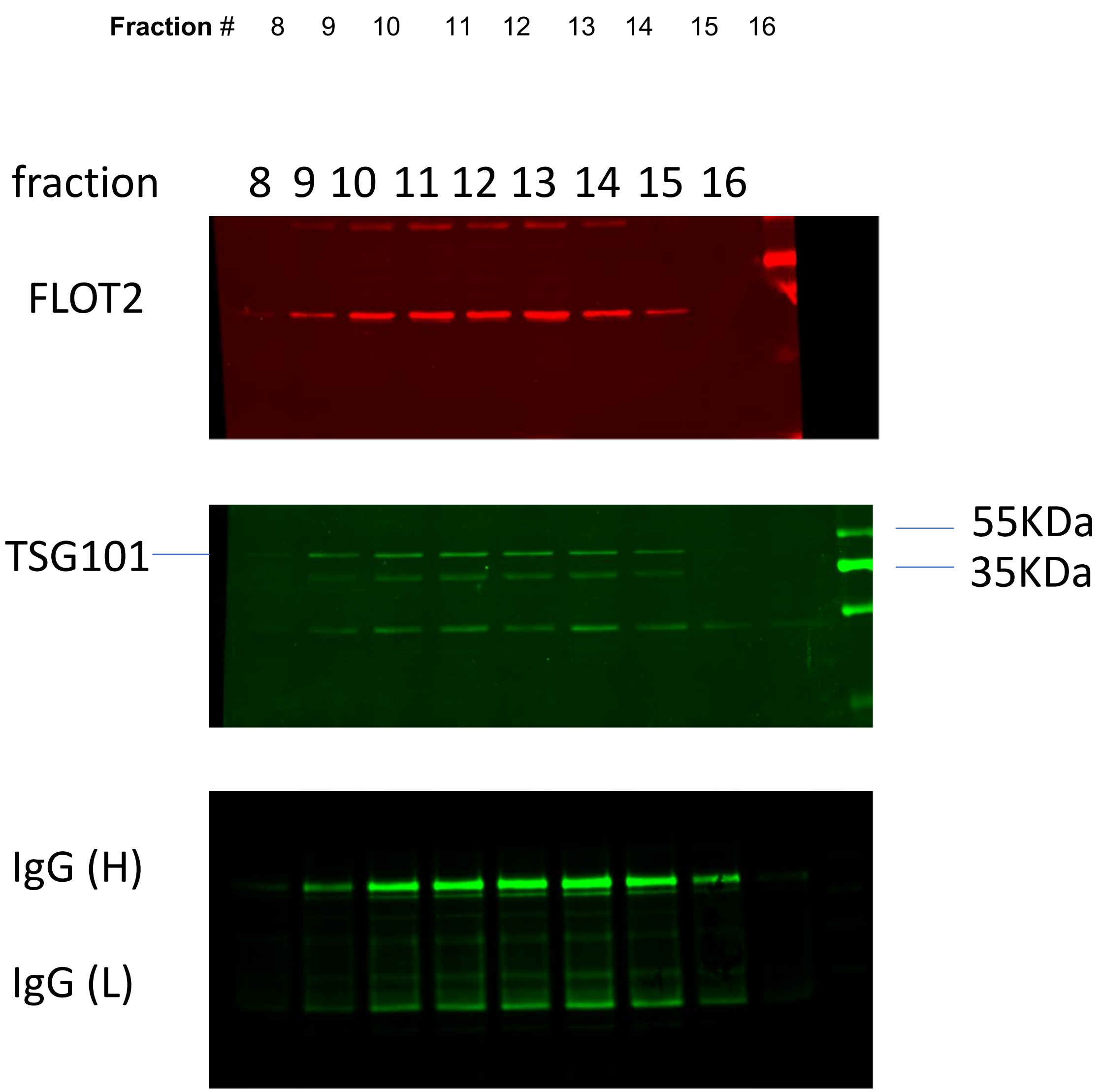

Fig 6 original scan
